# Prevention of medication related osteonecrosis of the jaw after dentoalveolar surgery: An institution’s experience

**DOI:** 10.4317/jced.56837

**Published:** 2020-08-01

**Authors:** Onur Şahin, Birkan Tatar, Ceren Ekmekcioğlu, Toghrul Aliyev, Onur Odabaşı

**Affiliations:** 1 Department of Oral and Maxillofacial Surgery, Faculty of Dentistry, İzmir Katip Çelebi University, İzmir, Türkiye; 2 Department of Oral and Maxillofacial Surgery, Faculty of Dentistry, Ankara Yıldırım Beyazıt University Ankara, Türkiye

## Abstract

**Background:**

Dentoalveolar surgery is a predisposing factor for medication related osteonecrosis of the jaw (MRONJ). The aim of our study was to evaluate the described surgical procedures to prevent the development of MRONJ after dentoalveolar surgery in patients receiving bisphosphonates.

**Material and Methods:**

In this retrospective study, sixty-three dentoalveolar surgeries were performed on 44 patients taking bisphosphonate in accordance with the treatment procedures we described. The following procedures were applied to patients 1) use of antibiotics 2) performed dentoalveolar surgical procedures 3) fill the socket with leukocyte- and platelet-rich fibrin (L-PRF) 4) post-operative application of low level laser therapy through Nd: YAG laser 5) sutures were removed on post-op 14th day 6) long-term results were evaluated.

**Results:**

Healing of all patients was uneventful. Complete mucosal healing was achieved in all patients at 1 month. There is no failure was observed in long-term follow-up.

**Conclusions:**

Because of the pathophysiology of MRONJ is not fully understood and has many risk factors, definitive protocols on prevention and treatment have not been established yet. Personal risk assessment is required for the prevention and treatment of MRONJ. The described surgical protocol may be considered to reduce the risk of developing MRONJ after dentoalveolar surgery due to its high success rate.

** Key words:**Tooth extraction, medication related osteonecrosis of the jaw, preventive dentistry, L-PRF, low level laser therapy.

## Introduction

Bisphosphonates (BPs) are the most commonly used antiresorptive drugs to prevent skeletal complications of many diseases (1). It is used to reduce serious complications such as hypercalcemia and pathological fractures, as well as to improve the quality of life and reduce pain complaints of cancer patients with bone metastases (2). One of the most important side effects of BPs is that it causes osteonecrosis in the jaw bones. Increased cases of osteonecrosis localized in the jaw bones related to BPs and antiresorptive drugs, negative impact on quality of life and increased morbidity led researchers to define early MRONJ and to investigate effective treatment modality. Although many studies have been conducted on the mechanism of action of bisphosphonates, its pathophysiology remains unclear. Bisphosphonates accumulate highly in bones and adjacent soft tissues with high turnover rates. Therefore, it is thought that if mucosal integrity is deteriorated, endothelial cell prophylaxis and wound healing delay and secondary infections occur in exposed mandible and maxilla, and later become osteonecrosis (3).

Several factors such as therapeutic indication, drug type and duration, local, demographic, systemic and genetic factors have been mentioned in determining the incidence of MRONJ (4). Local etiologic factors that are effective in the development of MRONJ are defined as dentoalveolar surgical procedures, anatomical factors, prosthesis and associated local oral inflammatory diseases. A large review by Filleul *et al.* (5) concluded that tooth extraction was the main triggering factor in 67% of MRONJ cases. Tooth extraction alone is a predisposing factor in MRONJ formation between 52% and 61% (6). The incidence of osteonecrosis after tooth extraction is reported to be 0.5% in patients using oral BPs, whereas it is reported to be between 1.6% and 14.8% in patients using BPs intravenously (7). These studies show that tooth extraction is an important risk factor for MRONJ formation. Therefore, various procedures have been determined to prevent the occurrence of osteonecrosis after tooth extraction. Studies include methods such as drug discontinuation, improved oral hygiene, antibiotic and mouthwash treatment, atraumatic extraction, primary closure of the extraction socket, laser biostimulation and use of autologous platelet concentrates (8,9).

The aim of our study was to evaluate the described surgical procedures to prevent the development of MRONJ after dentoalveolar surgery in patients receiving bisphosphonate therapy.

## Material and Methods

This retrospective study included 44 patients (32 females, 12 males) who received bisphosphonate and antiresorptive drug therapy and required dentoalveolar surgery between 2017 May-2019 May. The study was approved by the ethical committee of our university (Katip Çelebi University Non-Interventional Clinical Studies Institutional Review Board, Türkiye, No:311). All authors read the Helsinki Declaration and followed the guidelines in the study. Informed consent was obtained from all participants.

The study included patients who were received bisphosphonates for osteoporosis or oncologic purposes requiring dentoalveolar surgery. Patients with a history of radiotherapy, a history of metastasis localized to the jawbone and with MRONJ of the extraction site were excluded from the study.

-Procedure

The following procedures were applied to patients who referred to our department for dentoalveolar surgery.

**Figure 1:**
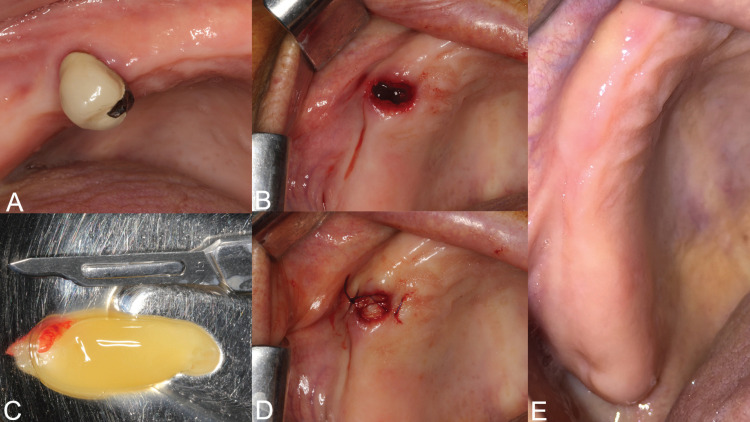
In the patient who has been using zoledronate for 6 years due to prostate cancer, upper right first premolar tooth with destructive tooth decay involving the roots was extracted. The prepared Leukocyte and platelet-rich fibrin (L-PRF) plug was laid directly over the bone to fill the socket. The wound edges were sutured with figure-of-eight sutures. No releasing vertical incisions or mucoperiosteal incisions were made for the primary closure. Complete mucosal healing was achieved at 1 month.

1) Demographic data, systemic diseases, history of bisphosphonate treatment (type of drug, route of use, duration of use), smoking habit, diabetes, steroid use were recorded at the first visit.

2) Clinical and radiological evaluation were performed to confirm the absence of MRONJ.

3) Patients continued to take medication.

4) 1000 mg of amoxicillin / clavulanic acid, 500 mg of metronidazole and 0.12% chlorhexidine digluconate mouthwash were prescribed for use 3 days before surgery and for 2 weeks post-op.

5) Surgical procedures were performed under local anesthesia (2 ml of 4% articaine hydrochloride with 1: 200,000 epinephrine). Intraligamental and intrapapillary anesthesia was not applied due not to prevent the recovery.

6) Only the sulcular incision was used in the extraction of the teeth with mucosal retention.

7) After the tooth extraction, the sharp bone edges were rounded with burs.

8) After the extraction, venous blood obtained from the patient’s peripheral blood was centrifuged at 3000 rpm for 10 minutes in 10 ml tubes without anticoagulant and obtained L-PRF placed into the extraction socket by Figure-of-eight sutures. No releasing vertical incisions or mucoperiosteal incisions were made for the primary closure.

9) Nd: YAG laser (Fotona-Slovenia) was used for biostimulation (wavelenght 1064 nm, power 1.25 W, frequency 15Hz, fiere 320 μmin diameter) on post-operative Day 2, 5, 7, 10, 14, 21 and 28 defocalised at 1-2 mm from the tissue for 1 min repeated 5 times.

10) Sutures were removed on post-op 14th day.

11) Patients using prosthesis discontinued to use their prosthesis for 3 months and then used with soft lining material.

12) Patients were examined clinically and radiologically at post op 1, 3 and 6 months. Figure 1 showed the surgical procedures for one of the patients.

The results were evaluated for wound healing. Treatment was considered successful when complete mucosal healing was achieved in the surgical field at the 1st and 3rd month controls without fistula or exposed bone symptoms. Data were collected retrospectively from patient records and surgical documents. For statistical analysis, group means and standard deviations were calculated for each variable ( age, gender, indication for BP, BP treatment duration and interval, BP type, route of drug administraiton,extraction site, treatment outcome and time of follow-up) by use of SPSS 21.0 (Statistical Package for the Social Sciences, Chicago, IL, USA). Chi-square test was used to calculate categorical variables and Student-t test was used to calculate quantitative variables. All data were evaluated at a signiﬁcance level of *p*< 0.05.

## Results

A total of 44 patients were included to the study. The subject group consisted of 32 females and 12 males and a mean age of 66.3. 44 patients underwent 63 dentoalveolar surgeries in accordance with the treatment procedures we described (Table 1). 21 of the patients were on medication for underlying malignant disease (14 patients had breast cancer, 6 patients had prostate cancer and 1 patient has nasopharynx cancer) and 23 patients were using medication for osteoporosis. 18 of the patients were on zoledronate, 8 patients were on ibandronate and 18 patients using alendronate. Mean duration of drug use via IV route: 44.6 months (25-108 months) and via oral route: 36.3 months (18-96 months). 23 of the dentoalveolar surgical sites were in the maxilla and 40 were in the mandible (Table 2). The response to treatment of each patient was recorded by regular controls. Healing of all patients was uneventful. Complete mucosal healing was achieved in all patients at 1 month (Fig. 1). The mean follow-up was 14.2 months (6-28 months). There was no significant difference between the each variable (age, gender, indication for BP, BP treatment duration and interval, BP type, extraction site) and in terms of healing time (*p*> 0.05).

**Table 1:**
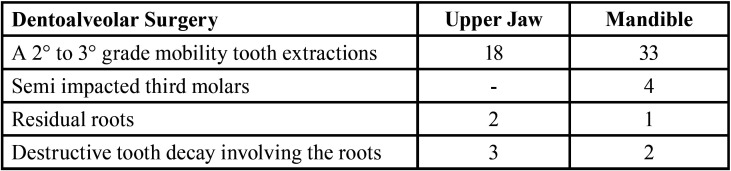
Dentoalveolar procedures performed in a group of patients.

**Table 2:**
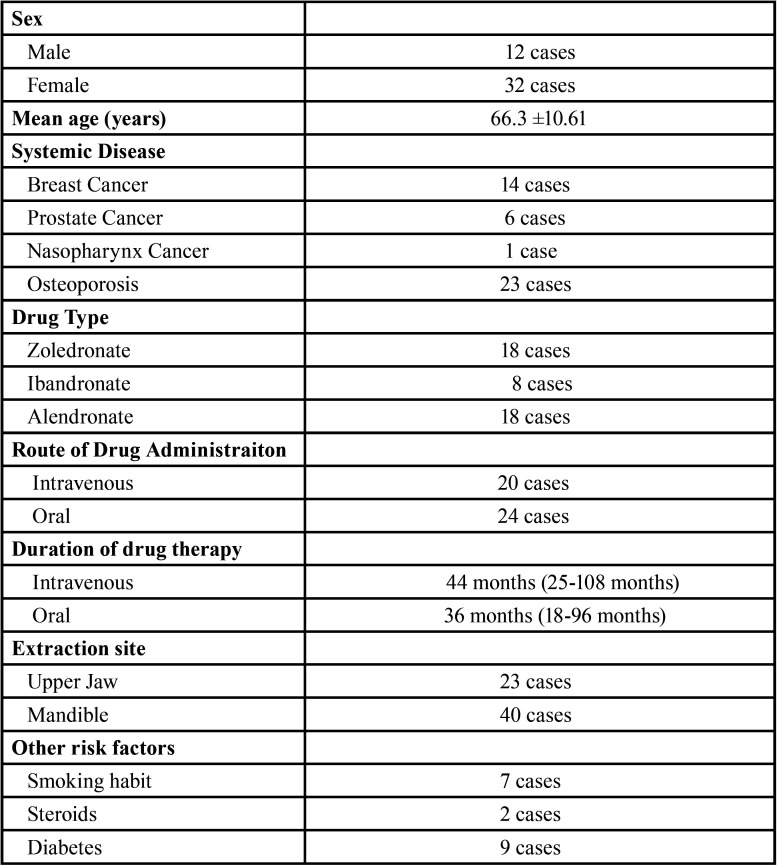
Clinical characteristics of patients included in the study.

## Discussion

BPs use increases day by day in the treatment of osteoporosis and slows down the resorption process in the bone. It is also used effectively in the treatment of multiple myeloma and metastatic bone tumors to improve the quality of life of the patient (10). However, the most serious side effect of these drugs is the risk of causing osteonecrosis, especially in the jaws (11). There is evidence to support that the incidence of MRONJ has been reduced by half in patients with preventive precautions before starting BP treatment (12). However, due to the lack of evidence-based studies in patients receiving bisphosphonate or antiresorptive therapy, a protocol to prevent MRONJ has not been established and is limited to the clinical experience of the researchers alone (13). Therefore, the aim of this study was to define a procedure to reduce the risk of MRONJ and stimulate healing after tooth extraction and to evaluate its effectiveness.

In the literature, drug discontinuation before surgical procedures are discussed. Drug discontinuation may be a procedure to reduce the risk of MRONJ in patients with osteoporosis who do not require an urgent tooth extraction and can be discontinued with bisphosphonate therapy (14). In contrast, Hasegawa *et al.* (15) reported in a multicenter study that short-term drug withdrawal had no significant effect on the incidence of MRONJ. In our study, drug discontinuation was not applied due to the systemic diseases of patients and the long half-life of bisphosphonates.

Local infections alter the pH within the lesion, which is thought to be an important factor in the pathogenesis of jawbone osteonecrosis (16). Teeth that are indicated for extraction due to residual roots, periodontitis, pericoronitis, excessive caries are a local factor of infection and a risk factor for MRONJ (17). In this study, local infection site were eliminated by extracting teeth or roots. Antibiotics and antiseptic mouthwashes were used for pre op 3 days and post op 2 weeks before sutures were taken to prevent bacterial contamination of the bone and the surgical site and the underlying bone.

Sharp bone edges in the surgical site increase the risk of secondary perforation; this is supported by the finding that MRONJ develops in the areas where mucosal thinning occurs (18). Therefore, all sharp bone edges in the surgical site should be eliminated to minimize the risk of secondary perforation (8). Thin oral mucosa on the sharp bone edge can be damaged by traumatic irritation. For this reason, the sharp edges of the bones were rounded in our patients and it was recommended that patients who use prosthesis should not use their prosthesis for 3 months. Three months later, the prostheses were lined and the patients were followed up.

The mechanism of action of BPs in the extraction socket has been described as decreasing oral epithelial cell migration, increasing apoptosis, preventing oral mucosal wound healing and inhibition of osteclastic activity (19). Hikita *et al.* (20) investigated the effects of bisphosphonates on post-extraction socket healing in rats. Histological and morphometric data showed that inhibition of osteoclast activity delayed post-extraction alveolar healing in rats injected with bisphosphonate compared to control group rats. The first 7 days after dentoalveolar surgery appear to be the most critical period for the onset of inflammatory processes that prevent healing and induce osteonecrosis (21). Autologous platelet consantrates (APCs) contain many growth factors and immune system components to support the healing process. Several clinical studies have reported the possible benefits of APCs in the prevention and treatment of MRONJ (22). Scoletta *et al.* (23) and Mazzetto *et al.* (24) reported that they prevented the formation of osteonecrosis by placing plasma rich in growth factors (PRGF) in the socket and Asaka *et al.* (25) reported that they prevented the formation of osteonecrosis by placing PRF. PRF is a second generation platelet concentrate that regulates inflammation and stimulates chemotactic factors involved in the immune response. Unlike platelet rich plasma (PRP) and PRGF, it is a completely autogenous biomaterial obtained without adding any external substances (26).

In this study, we applied L-PRF to the extraction sockets simultaneously with tooth extraction in patients at risk of MRONJ. Osteonecrosis was not encountered in any of our patients during short and long term follow-up. Previous studies have highlighted the importance of primary closure of extraction sockets. Vertical incisions or mucoperiosteal incisions were not performed to prevent pain and edema. The wound edges were sutured with Figure-of-eight sutures.

The effects of low-level laser therapy (LLLT) on wound healing have been reported *in vitro* and *in vivo* studies as faster epithelialization with decreased inflammation and increased collagen and granulation tissue (27). In 2011, Kan *et al.* (28) reported that they used laser therapy to prevent BRONJ after tooth extraction. Vescovi *et al.* reported that they prevented BRONJ after tooth extraction in patients under BP treatment and in high-risk patients with a history of BRONJ, using a clinical protocol supported by Nd: YAG laser therapy in two separate studies (29,30). In our study, we used Nd: YAG laser for biostimulation in accordance with the protocol of Vescovi *et al.* (30).

## Conclusions

Because the pathophysiology of MRONJ is not fully understood and has many risk factors, definitive protocols on prevention and treatment have not been established yet. Personal risk assessment is required for the prevention and treatment of MRONJ. The surgical protocol presented in this study shows promising results for MRONJ protection after dentoalveolar surgery. Although prospective studies with larger sample sizes are needed, we believe that our study will lead to better designed future studies.
